# A computational clinical decision-supporting system to suggest effective anti-epileptic drugs for pediatric epilepsy patients based on deep learning models using patient’s medical history

**DOI:** 10.1186/s12911-024-02552-w

**Published:** 2024-05-31

**Authors:** Daeahn Cho, Myeong-Sang Yu, Jeongyoon Shin, Jingyu Lee, Yubin Kim, Hoon-Chul Kang, Se Hee Kim, Dokyun Na

**Affiliations:** 1 Department of Biomedical Engineering, Chung-Ang University, 84 Heukseok-ro, Dongjak-gu, Seoul, Republic of Korea; 2 Division of Pediatric Neurology, Department of Pediatrics, Severance Children’s Hospital, Yonsei University College of Medicine, Epilepsy Research Institute, 50-1 Yonsei-ro Seodaemun-Gu, Seoul, Republic of Korea

**Keywords:** Clinical decision-supporting system, Pediatric Epilepsy, Anti-epileptic drug, Medical history, Convolutional neural network, Machine learning

## Abstract

**Background:**

Epilepsy, a chronic brain disorder characterized by abnormal brain activity that causes seizures and other symptoms, is typically treated using anti-epileptic drugs (AEDs) as the first-line therapy. However, due to the variations in their modes of action, identification of effective AEDs often relies on ad hoc trials, which is particularly challenging for pediatric patients. Thus, there is significant value in computational methods capable of assisting in the selection of AEDs, aiming to minimize unnecessary medication and improve treatment efficacy.

**Results:**

In this study, we collected 7,507 medical records from 1,000 pediatric epilepsy patients and developed a computational clinical decision-supporting system for AED selection. This system leverages three multi-channel convolutional neural network (CNN) models tailored to three specific AEDs (vigabatrin, prednisolone, and clobazam). Each CNN model predicts whether a respective AED is effective on a given patient or not. The CNN models showed AUROCs of 0.90, 0.80, and 0.92 in 10-fold cross-validation, respectively. Evaluation on a hold-out test dataset further revealed positive predictive values (PPVs) of 0.92, 0.97, and 0.91 for the three respective CNN models, representing that suggested AEDs by our models would be effective in controlling epilepsy with a high accuracy and thereby reducing unnecessary medications for pediatric patients.

**Conclusion:**

Our CNN models in the system demonstrated high PPVs for the three AEDs, which signifies the potential of our approach to support the clinical decision-making by assisting doctors in recommending effective AEDs within the three AEDs for patients based on their medical history. This would result in a reduction in the number of unnecessary ad hoc attempts to find an effective AED for pediatric epilepsy patients.

## Background

Epilepsy is an intractable chronic neurological disease that involves abnormal brain activity such as seizures and other symptoms, posing significant challenges to patients worldwide. According to the World Health Organization, approximately 50 million individuals worldwide are suffering from epilepsy, with an estimated five million new diagnoses annually [1]. Notably, in the United States alone, there are three million adult epilepsy patients and 470,000 pediatric epilepsy patients [2].

Generally, anti-epileptic drugs (AEDs) serve as a primary therapy for symptom management in the majority of epilepsy patients [3]. However, it is often necessary to adopt an ad hoc trial-and-error approach to identify effective AEDs due to the diverse causes of epilepsy and the variations of AEDs in their modes of action [4, 5]. Such empirical strategies may have a detrimental effect on the quality of life provided to patients, particularly pediatric epilepsy patients, due to the inherent risks and potential adverse effects associated with clinical trials [6−9]. Therefore, the minimization of the trials to find out effective AEDs is crucial in epilepsy treatment and there is a continuous demand for computational methods to suggest an effective AED for pediatric patients, minimizing the ad hoc trials.

With the rapid advance of machine learning methods, such as convolutional neural network (CNN) [10] and long short-term memory network [11], there is a newfound opportunity to develop computational methods for predicting individual seizure occurrences and surgical outcomes based on medical data, comprising primarily textual and visual information [12−15]. However, there are few studies on precise patient-specific drug suggestion methods for epilepsy patients, and there have been no studies that develop personalized drug suggestion models for pediatric epilepsy patients [16−18]. Previous studies have utilized electronic health records (EHR) and genomic data to predict drug outcomes [16−18]. However, the high costs associated with genetic testing present a significant financial barrier to the practical application of these models for the selection of personalized drugs [19, 20]. Although EHR-based drug recommendation approach does not mandate additional costly tests, the reported performances, with F1-scores ranging from 0.20 to 0.40, undermine its practical utility [16]. Consequently, despite the previous studies on patient-specific drug suggestion methods, there is a continued demand for cost-effective and precise methods.

In this study, we developed a computational clinical decision-supporting system tailored for pediatric epilepsy patients based on their medical history. Our system relies on three multi-channel CNN models dedicated to vigabatrin, prednisolone, and clobazam, respectively. These CNN models in our system predict the efficacy of specific AEDs for a given pediatric epilepsy patient based solely on medical history, encompassing prescription histories and EEG interpretation reports. This signifies the potential of our system to support doctors in prescribing effective and personalized AEDs for individual patients, thereby enhancing clinical decision-making processes in the treatment of pediatric epilepsy.

## Results and discussion

### Aim of study

The primary objective of this study is to develop a system that assists doctors in making clinical decisions regarding the AED selection for pediatric epileptic patients based on their medical history. The system conducts a comprehensive analysis of each patient’s medical history and provides recommendations on the potentially effective AEDs based on the predictions of effectiveness of vigabatrin, prednisolone, and clobazam. The system identifies patterns within the medical history associated with the efficacy of AED, thereby providing suggestions about personalized and optimal AED for individual patients.

### Patient data analysis and processing

**Fig. 1:**
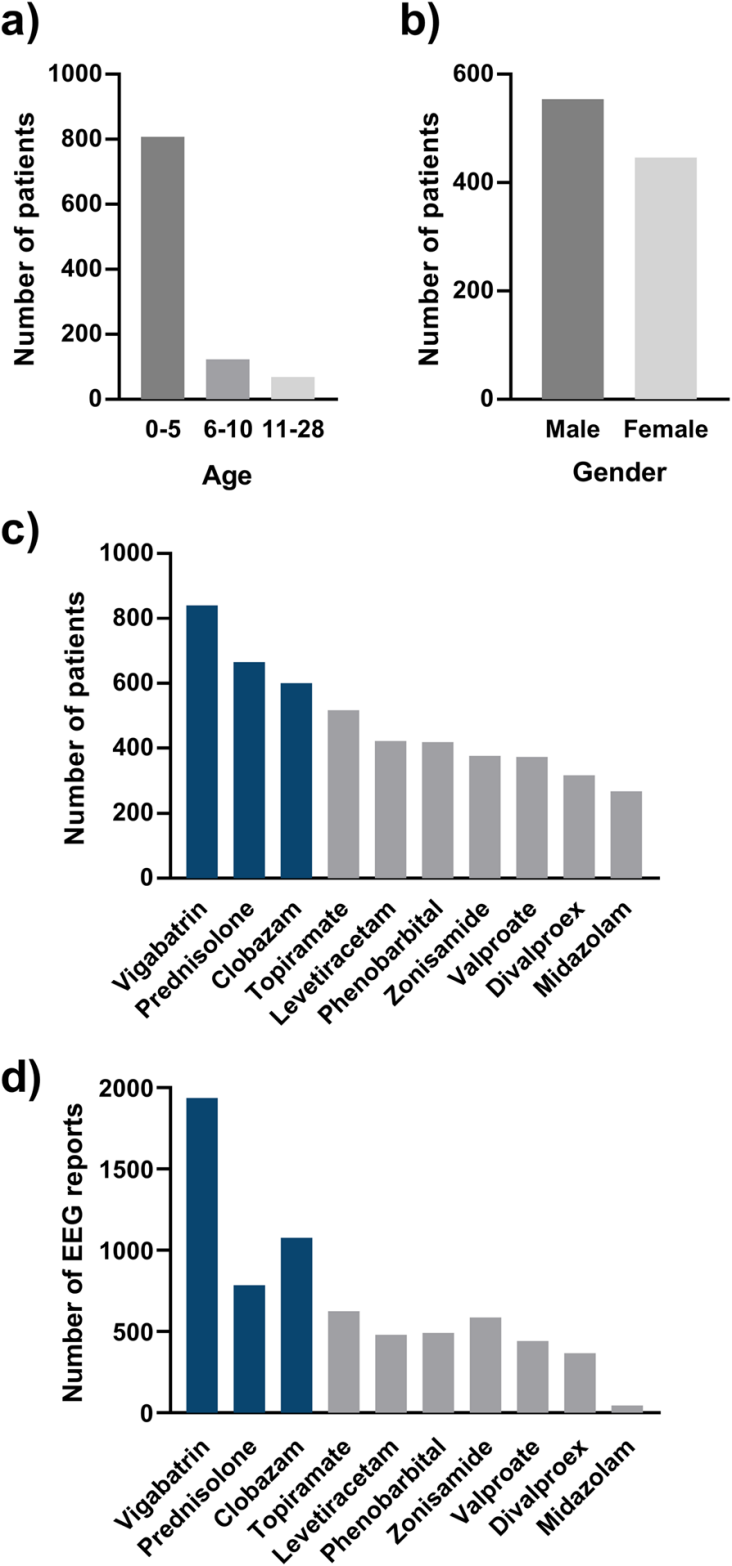
The data distribution of age, gender, and AED. **a** The distribution of patient age. **b** The distribution of patient gender. **c** The number of patients prescribed each AED. **d** The number of EEG interpretation reports corresponds to the ten most frequently prescribed AEDs. In (c) and (d), the top ten highly prescribed AEDs are shown and the target three AEDs of our drug suggestion system are colored in blue

We collected the medical history data of 1,000 pediatric epilepsy patients from the Epilepsy Research Institute, Severance Children’s Hospital, Republic of Korea. Figure 1 presents the statistics of the compiled medical data, which includes age, gender, prescribed AEDs, and EEG interpretation reports. A significant proportion of the patients, 81%, were notably within the age of zero to five years (Fig. 1a). The rest, 19%, were aged between six and 28 years. As for the gender distribution, it was balanced with 554 males and 446 females (Fig. 1b).

A total of 52 AEDs were prescribed to the 1,000 pediatric epilepsy patients, and 7,507 post-medication EEG interpretation reports were collected. Among these AEDs, the ten most frequently prescribed drugs were vigabatrin (840 patients with 1,937 EEG reports), prednisolone (665 patients with 785 EEG reports), clobazam (600 patients with 1,078 EEG reports), topiramate (517 patients with 625 EEG reports), levetiracetam (422 patients with 481 EEG reports), phenobarbital (419 patients with 492 EEG reports), zonisamide (376 patients with 588 EEG reports), valproate (373 patients with 443 EEG reports), divalproex (316 patients with 368 EEG reports), and midazolam (267 patients with 46 EEG reports) (Fig. 1c and d).

Given the necessity for sufficient training data to develop accurate machine learning models, it was observed that the majority of drugs in our dataset lacked sufficient quantification in terms of both prescribed patients and medical data. As a result, due to the limited availability of data, only the three most frequently prescribed AEDs (vigabatrin, prednisolone, and clobazam) were suitable for the model construction. Thus, we focused on developing individual CNN models dedicated to predicting the efficacy of each of those AEDs for pediatric epilepsy patients. Specifically, counts of EEG interpretation reports for these AEDs were 1,937 (70 relieved and 1,867 non-relieved), 784 (15 relieved and 770 non-relieved), and 1,078 (18 relieved and 1,060 non-relieved), respectively (Table 1). As epilepsy is a seizure disease, there are diverse types of epilepsy and, in certain people, their causes are not known yet, which makes ad hoc trials and errors inevitable in the search for effective AEDs. Therefore, less than 5% of patients experienced symptom relief after medication, leading to highly imbalanced datasets (Table 1).

**Table 1:** The number of patients who were prescribed an AED and the corresponding EEG interpretation reports

AEDs	Patients	EEG interpretation reports	
		Relieved	Non-relieved
Vigabatrin	840	70	1867
Prednisolone	665	15	770
Clobazam	660	18	1060

**Fig. 2:**
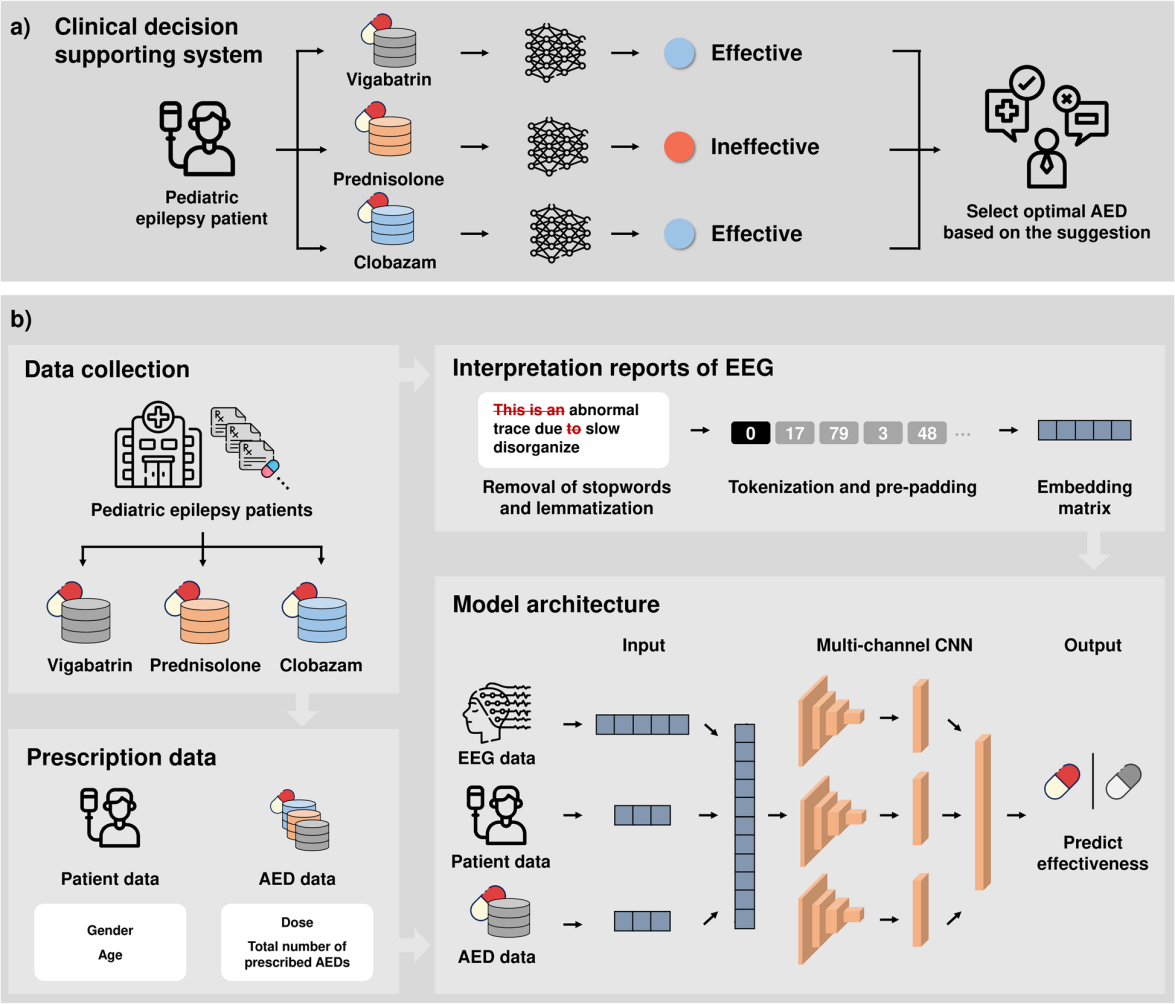
The schematic illustration of our clinical decision-supporting system. Our clinical decision-supporting system is comprised of three multi-channel CNN models, which predict the outcome of respective AED: vigabatrin, prednisolone, and clobazam. **a** The comprehensive overview of our clinical decision-supporting system. **b** The processes of pediatric epilepsy patient data collection and construction of the datasets, generation of an embedding matrix using an interpretation report of EEG, and architecture of our CNN models in the clinical decision-supporting system

As illustrated in Fig. 2, the collected data comprised various types, including textual (EEG interpretation reports), numerical (age, total number of prescribed AEDs and their doses), and categorical (gender) data. The numerical and categorical data were directly utilized as features, while the textual data underwent preprocessing for featurization. The featurization involved several steps: extraction of redundant terms from EEG interpretation reports using WordNetLemmatizer from the Natural Language Toolkit (NLTK) [21], encoding of each lemma into a numerical 1D vector through Tokenizer [22], pre-padding of vectors with zeros to maintain consistency, and mapping of padded vectors to pretrained GloVe word embedding for semantic enrichment [23]. Subsequently, the EEG interpretation reports were transformed into embedding matrices tailored to vigabatrin, prednisolone, and clobazam, respectively, with dimensions 194, 190, and 208. These embedding matrices served as the input features for corresponding CNN models.

### Model construction

Given that our target AEDs consisted of three drugs, we considered two approaches: developing a single multi-class model to predict the efficacy of all three AEDs simultaneously or developing three independent binary classification models, each dedicated to predicting the efficacy of a single AED. However, due to the limited number of patients prescribed all three AEDs (only 103 patients), there was insufficient data to construct a robust multi-class model. Consequently, three distinct multi-channel CNN models were constructed, each tailored to a single AED. These models formed the foundation of our AED suggestion system (Fig. 2a).

The architectural overview of our models is illustrated in Fig. 2b. Briefly, as described in Methods, each CNN model features dedicated channels responsible for extracting features from the patient’s medical history data. These features are then aggregated via max-pooling, combined, flattened, and subsequently passed through a dropout layer equipped with *softmax* activation to predict the efficacy of a specific AED for the queried patient. As a result, our decision-supporting system comprises three individual CNN models, with each model predict the potential efficacy of single AED for a given patient. During a hyperparameter tuning phase, we optimized several parameters as follows (Table 2): the number of channels was set to three, with corresponding filter sizes of three, four, and five. A dropout value of 0.5 was employed, coupled with a learning rate of 0.001 and an epoch size of 100. Furthermore, batch sizes were set to 128, 32, and 32 for vigabatrin, prednisolone, and clobazam, respectively.

**Table 2:** The ranges of parameters in hyperparameter optimization

Parameters	Range
Number of channels	[1, 2, 3, 4, 5]
Filter size	[2, 3, 4, 5, 6, 7, 8, 10]
Values of dropout	[0.2, 0.3, 0.4, 0.5]
Learning rate	[0.0005, 0.0001, 0.005, 0.001, 0.05, 0.01]
Epochs	[32, 50, 64, 100, 128, 150]
Batch sizes	[16, 32, 64, 128]

### Prediction performance

We conducted ten iterations of 10-fold cross-validation and performance assessment on a hold-out dataset to ensure robustness. For the comparison with other algorithms, we constructed models employing conventional algorithms such as k-nearest neighbors (KNN), logistic regression, naïve Bayes, random forest, and LightGBM. This comparison was crucial due to the relatively small patient population in our dataset, where conventional machine learning algorithms might exhibit superior performances compared with advanced deep learning algorithms.

The results of 10-fold cross-validation are shown in Table 3. Our CNN models for vigabatrin, prednisolone, and clobazam achieved AUROCs of 0.90, 0.80, and 0.92, respectively, along with positive predictive values (PPVs) of 0.94, 0.91, and 0.90. Notably, our CNN models consistently outperformed other models. For instance, KNN models achieved AUROCs of 0.79, 0.67, and 0.67, and LightGBM achieved AUROCs of 0.82, 0.61, and 0.69 for the three AEDs, respectively. The validation results on hold-out test datasets, as shown in Table 4, demonstrated the superiority of our CNN models. Across the all metrics, our models showed either better or comparable performance. Notably, the PPVs of our models were 0.92, 0.97, and 0.91 for the three AEDs, while those of KNN were 0.60, 0.50, and 0.35, and LightGBM resulted in 0.65, 0.30, and 0.50, respectively.

**Table 3:** The results of 10-fold cross-validation of our models and conventional machine learning models for each AED.

AEDs	Models	AUROC	BAL-ACC	SEN	SPE	PPV	NPV
Vigabatrin	Our model	0.90 ± 0.003	0.93 ± 0.030	0.88 ± 0.057	0.99 ± 0.003	0.94 ± 0.037	1.00 ± 0.000
	KNN	0.79 ± 0.024	0.88 ± 0.025	0.78 ± 0.047	0.98 ± 0.003	0.58 ± 0.047	1.00 ± 0.000
	Logistic regression	0.77 ± 0.015	0.60 ± 0.006	0.22 ± 0.009	0.99 ± 0.003	0.66 ± 0.037	0.89 ± 0.006
	Naïve Bayes	0.74 ± 0.022	0.54 ± 0.003	0.10 ± 0.003	0.99 ± 0.003	0.77 ± 0.047	0.72 ± 0.015
	Random forest	0.81 ± 0.028	0.83 ± 0.052	0.68 ± 0.101	0.99 ± 0.003	0.65 ± 0.056	0.98 ± 0.009
	LightGBM	0.82 ± 0.028	0.92 ± 0.029	0.85 ± 0.056	0.99 ± 0.003	0.65 ± 0.056	0.99 ± 0.003
Prednisolone	Our model	0.80 ± 0.047	0.92 ± 0.031	0.85 ± 0.063	1.00 ± 0.000	0.91 ± 0.126	1.00 ± 0.000
	KNN	0.67 ± 0.072	0.83 ± 0.036	0.68 ± 0.069	0.99 ± 0.003	0.50 ± 0.142	1.00 ± 0.000
	Logistic regression	0.63 ± 0.069	0.65 ± 0.052	0.31 ± 0.101	0.99 ± 0.003	0.35 ± 0.142	0.92 ± 0.012
	Naïve Bayes	0.62 ± 0.072	0.52 ± 0.004	0.06 ± 0.012	0.99 ± 0.003	0.40 ± 0.126	0.78 ± 0.018
	Random forest	0.70 ± 0.069	0.53 ± 0.015	0.08 ± 0.028	0.99 ± 0.003	0.55 ± 0.148	0.85 ± 0.037
	LightGBM	0.61 ± 0.063	0.52 ± 0.015	0.07 ± 0.027	0.98 ± 0.003	0.30 ± 0.126	0.91 ± 0.012
Clobazam	Our model	0.92 ± 0.050	0.91 ± 0.045	0.82 ± 0.088	1.00 ± 0.003	0.90 ± 0.101	1.00 ± 0.000
	KNN	0.67 ± 0.069	0.82 ± 0.064	0.65 ± 0.126	0.99 ± 0.003	0.35 ± 0.142	1.00 ± 0.00
	Logistic regression	0.64 ± 0.069	0.68 ± 0.018	0.38 ± 0.034	0.99 ± 0.003	0.35 ± 0.142	0.98 ± 0.006
	Naïve Bayes	0.57 ± 0.066	0.50 ± 0.004	0.02 ± 0.006	0.99 ± 0.003	0.45 ± 0.148	0.69 ± 0.006
	Random forest	0.70 ± 0.072	0.56 ± 0.031	0.14 ± 0.060	0.99 ± 0.003	0.50 ± 0.142	0.91 ± 0.015
	LightGBM	0.69 ± 0.069	0.54 ± 0.040	0.09 ± 0.037	0.99 ± 0.003	0.50 ± 0.142	0.88 ± 0.027

* Mean ± standard error

**Table 4:** Performance test results of our models and models trained using conventional algorithms

AEDs	Models	BAL-ACC	SEN	SPE	PPV	NPV
Vigabatrin	Our model	0.94 ± 0.007	0.89 ± 0.012	0.99 ± 0.003	0.92 ± 0.012	0.99 ± 0.003
	KNN	0.83 ± 0.020	0.68 ± 0.037	0.98 ± 0.003	0.60 ± 0.069	1.00 ± 0.003
	Logistic regression	0.60 ± 0.006	0.22 ± 0.009	0.99 ± 0.003	0.68 ± 0.066	0.91 ± 0.006
	Naïve Bayes	0.54 ± 0.003	0.10 ± 0.003	0.99 ± 0.003	0.73 ± 0.053	0.75 ± 0.012
	Random forest	0.83 ± 0.052	0.68 ± 0.101	0.99 ± 0.003	0.65 ± 0.056	0.98 ± 0.009
	LightGBM	0.92 ± 0.059	0.85 ± 0.056	0.99 ± 0.003	0.65 ± 0.056	0.99 ± 0.003
Prednisolone	Our model	0.95 ± 0.028	0.92 ± 0.053	0.99 ± 0.003	0.97 ± 0.006	0.99 ± 0.003
	KNN	0.78 ± 0.037	0.58 ± 0.072	0.99 ± 0.003	0.50 ± 0.142	1.00 ± 0.000
	Logistic regression	0.65 ± 0.052	0.31 ± 0.101	0.99 ± 0.003	0.50 ± 0.142	0.98 ± 0.003
	Naïve Bayes	0.52 ± 0.007	0.06 ± 0.012	0.99 ± 0.003	0.70 ± 0.126	0.82 ± 0.012
	Random forest	0.53 ± 0.031	0.08 ± 0.028	0.99 ± 0.003	0.55 ± 0.148	0.85 ± 0.037
	LightGBM	0.52 ± 0.031	0.07 ± 0.028	0.98 ± 0.003	0.30 ± 0.126	0.91 ± 0.012
Clobazam	Our model	0.92 ± 0.014	0.85 ± 0.025	0.99 ± 0.003	0.91 ± 0.006	0.97 ± 0.006
	KNN	0.80 ± 0.029	0.62 ± 0.056	0.99 ± 0.003	0.35 ± 0.142	1.00 ± 0.000
	Logistic regression	0.53 ± 0.018	0.08 ± 0.034	0.99 ± 0.003	0.35 ± 0.142	0.93 ± 0.006
	Naïve Bayes	0.50 ± 0.004	0.02 ± 0.006	0.99 ± 0.003	0.45 ± 0.148	0.69 ± 0.041
	Random forest	0.56 ± 0.031	0.14 ± 0.060	0.99 ± 0.003	0.50 ± 0.142	0.91 ± 0.015
	LightGBM	0.54 ± 0.020	0.09 ± 0.037	0.99 ± 0.003	0.50 ± 0.142	0.88 ± 0.028

* Mean ± standard error

In this study, as the primary goal is to suggest the efficacies of AEDs through the system, particular emphasis is placed on PPVs. PPV holds paramount importance in clinical decision-supporting systems, as it directly reflects the accuracy of drug suggestion and determines the practical applicability of the model in making clinical decisions [24]. Given that our system aimed to support clinical decisions by predicting the drug outcomes of three AEDs, PPV is a critical metric. Our CNN models achieved exceptionally high PPVs exceeding 0.9, as listed in Tables 3 and 4. This highlights their effectiveness in identifying effective AEDs. In contrast, conventional models achieved lower PPVs. This contrast underscores the practical applicability of our clinical decision-supporting system, built upon three multi-channel CNN models, in the field of clinical decision-supporting. The high PPVs enable our system to assist the doctors in selecting an effective AED, thereby reducing the need for unnecessary trial and error in drug selection processes.

### Limitations of our models

Current drug suggestion models often rely on costly techniques such as whole-genome sequencing, which are not feasible for many healthcare settings [16−18]. Similarly, other omics data such as transcriptomic and proteomic data face similar limitations due to their associated costs [25]. To overcome these limitations, we have proposed a computational clinical decision-supporting system based on deep learning models that utilize patient medical history data. This offers a more practical and cost-effective alternative to expensive omics experiments. In addition to the practical implications, we have demonstrated the effectiveness of the multi-channel CNN method in extracting valuable information from unstructured textual data for prediction tasks.

Although our CNN models showed high accuracy in predicting drug efficacies for the three AEDs, they were developed from limited and highly imbalanced datasets for pediatric epilepsy patients. There are many other AEDs (52 AEDs in our collected dataset) that were not sufficiently prescribed in our dataset, which prevented us from building models for the remaining 49 AEDs. Additionally, due to variations in the mode of action among AEDs, effective AEDs for individuals are scarce, resulting in highly imbalanced datasets. Finally, since our focus was on pediatric epilepsy patients, the number of patients was significantly lower. To develop more reliable and accurate prediction models covering all available AEDs, a larger patient dataset is necessary, which could be achieved through collaboration across many hospitals worldwide.

## Conclusion

Our study presents a novel computational clinical decision-supporting system designed to assist the personalized selection of AEDs for pediatric patients. Using multi-channel CNN models and patient medical history data, our system offers a practical and cost-effective alternative to existing methodologies, which often rely on expensive techniques such as whole-genome sequencing. The analysis and validation of our models have demonstrated the effectiveness of our approach in identifying effective AEDs, as evidenced by high PPVs exceeding 0.9. Despite the challenges associated with limited patient data and specific AEDs, our system demonstrated its robustness and potential, paving the way for the future advancements in the field of personalized AED selection. In conclusion, our study contributes to the enhancement of clinical decision-making processes in the treatment of pediatric epilepsy patients and holds promise for the improvement of treatment processes with the minimization of unnecessary trials in drug selection.

## Methods

### Data preparation

The medical history and drug response dataset of 1,000 pediatric epilepsy patients was obtained from the Epilepsy Research Institute, Severance Hospital in Seoul, Republic of Korea. The data was collected between 2010 and 2021, and 808 patients were aged between zero and five years. The data includes prescription records for 52 AEDs and 7,507 medical reports. The following data were extracted from patient information and medical reports: age, gender, dose of prescribed AED, total number of prescribed AEDs, EEG interpretation report, and EEG impression report.

The EEG impression report was a crucial determinant in assessing whether a prescribed AED alleviated the epileptic symptoms. It was used exclusively for this determination. For this assessment, we analyzed EEG impression reports that were documented within a maximum duration of a month post-prescription. If the impression report indicated normalized EEG patterns or symptom relief post-medication, the prescribed AED was classified as effective. Conversely, if such improvements were not observed, the AED was categorized as ineffective. In the development of our models, we utilized effective AEDs as positive instances and ineffective AEDs as negative instances.

### Data preprocessing and data embedding

Features extracted from medical history were categorized into three groups based on their data types: textual data (EEG interpretation report), numerical data (age, total number of prescribed AEDs and their doses), and categorical data (gender). The EEG interpretation reports were written in a freeform text by doctors. However, the text was unstructured and contained numerous stopwords, such as ‘a’, ‘an’, and ‘the’, potentially introducing bias into the model [26, 27]. To address this issue, the textual data was preprocessed using NLP methods such as WordNetLemmatizer of NLTK and Tokenizer of Keras [21, 22]. For instance, “This is an abnormal tracing due to slow disorganized background rhythm.” would be transformed into “abnormal trace due slow disorganize background rhythm” after lemmatization and removal of stopwords such as “This”, “is”, “an”, and “to”. Subsequently, the processed sentence would be tokenized, resulting in a sequence vector such as [17, 79, 3, 48, 23, 7, 10]. Given the variable length of textual data, the tokenized text would be pre-padded with zeros to maintain consistency, resulting in a 1D vector representation of the textual data (e.g. [0, 17, 79, 3, 48, 23, 7, 10]). These preprocessing steps ensured the conversion of text into a machine-interpretable format. Furthermore, during the preprocessing, the lemmatization and removal of stopwords enhanced the quality of the textual features, and the preprocessed textual features were subsequently transformed into matrices using precomputed GloVe embedding [23].

### Construction of clinical decision-supporting system

We developed three multi-channel CNN models as part of a clinical decision-supporting system, each dedicated to predicting the effectiveness of a specific AED (vigabatrin, prednisolone, and clobazam). Inspired by the success of CNNs in various fields, such as image and text processing, we adapted CNN for medical text classification, using its inherent ability to learn features from text data in a position-independent manner [28−30]. By treating text as an image and employing 1D vector, CNN can capture meaningful word combinations regardless of their position. This automatic feature extraction enables the identification of complex relationships and enhances accuracy, even with varying sentence structures.

Our CNN models employed three filters of different sizes to extract various features. Each model comprised three different convolutional layers and corresponding max-pooling layers. The features extracted by these convolutional and pooling layers were then concatenated and flattened into a 1D vector, which was subsequently processed by a dropout layer to prevent overfitting. The flattened feature vector was passed to a fully connected output layer with a *softmax* activation function to predict the effectiveness of the AED.

In order to train the respective CNN models, the dataset was divided into a training set and a testing set in a 9:1 ratio, respectively. Various performance metrics, including AUROC, PPV, negative predictive value (NPV), sensitivity (SEN), and specificity (SPE), were calculated. To address the imbalance of the dataset, each class in the dataset was weighted based on the number of samples and balanced accuracy (BAL-ACC) was calculated in order to provide a more robust metric of the predictive performances [31]. The data splitting and evaluation processes were repeated ten times to ensure the robustness of the models. Both averaged performance metrics and standard errors were calculated *via* iteration. Additionally, hyperparameters were optimized *via* 10-fold cross-validation, and the ranges of the hyperparameters are listed in Table 2.

### Other machine learning models

To facilitate a comprehensive comparison, five additional models were constructed using widely employed conventional machine learning algorithms, including KNN, logistic regression, naïve Bayes, random forest, and LightGBM [32]. These models were built using the same features and the dataset utilized for our CNN models, ensuring fair comparison and performance across different algorithms. The ten times iteration process of data splitting and evaluation was conducted in the same manner as for our CNN models. The same metrics were calculated consistently to evaluate the predictive performances of the models and enable the comparison with our CNN models.

## Abbreviations

AED: Anti-epileptic drug
AUROC: Area under the receiver-operating characteristic curve
BAL-ACC: Balanced accuracy
CNN: Convolutional neural network
EEG: Electroencephalogram
EHR: Electronic health record
KNN: k-nearest neighbors
NLP: Natural language processing
NLTK: Natural language toolkit
NPV: Negative predictive value
PPV: Positive predictive value
SEN: Sensitivity
SPE: Specificity
